# Utility of narrow-band imaging plus texture and color enhancement imaging mode in the evaluation of a rectal superficially serrated adenoma overlying a surgical scar

**DOI:** 10.1055/a-2938-1586

**Published:** 2026-10-01

**Authors:** Takehiro Yoshii, Naoya Toyoshima, Ryosuke Kawagoe, Yasuhiko Mizuguchi, Katsura Nakayama, Toshihiro Haga, Yutaka Saito

**Affiliations:** 1 Endoscopy Division 68380 National Cancer Center HospitalTokyoTokyoJapan; 2 Department of Pathology 68380 National Cancer Center HospitalTokyoTokyoJapan

Texture and color enhancement imaging (TXI) improves the detection of colorectal lesions. 1​ Superficially serrated adenomas typically present as flat mucosal elevations with clear margins. 2​ However, their demarcation can be obscured, particularly in the lower rectum (Rb). The “skirt” finding—a flat, pale extension at the tumor margin—contributes to these unclear margins. 3​

A 47-year-old woman presented with a 25-mm 0-IIa lesion in the Rb. Magnifying narrow-band imaging (NBI) demonstrated a JNET Type 2A pattern, and chromoendoscopy revealed a Type IVH pit pattern. Notably, this lesion overlapped a previous hemorrhoidectomy scar ( Fig 1 ). Due to the skirt finding, severe mucosal fibrosis and isochromic background changes, the tumor's demarcation line (DL) was obscured under conventional white-light imaging and standard NBI. Indigo carmine spraying made the borders of the flat elevation distinct, but margin delineation of the flat component near the scar was difficult ( Fig 2 ).

**Fig. 1:**
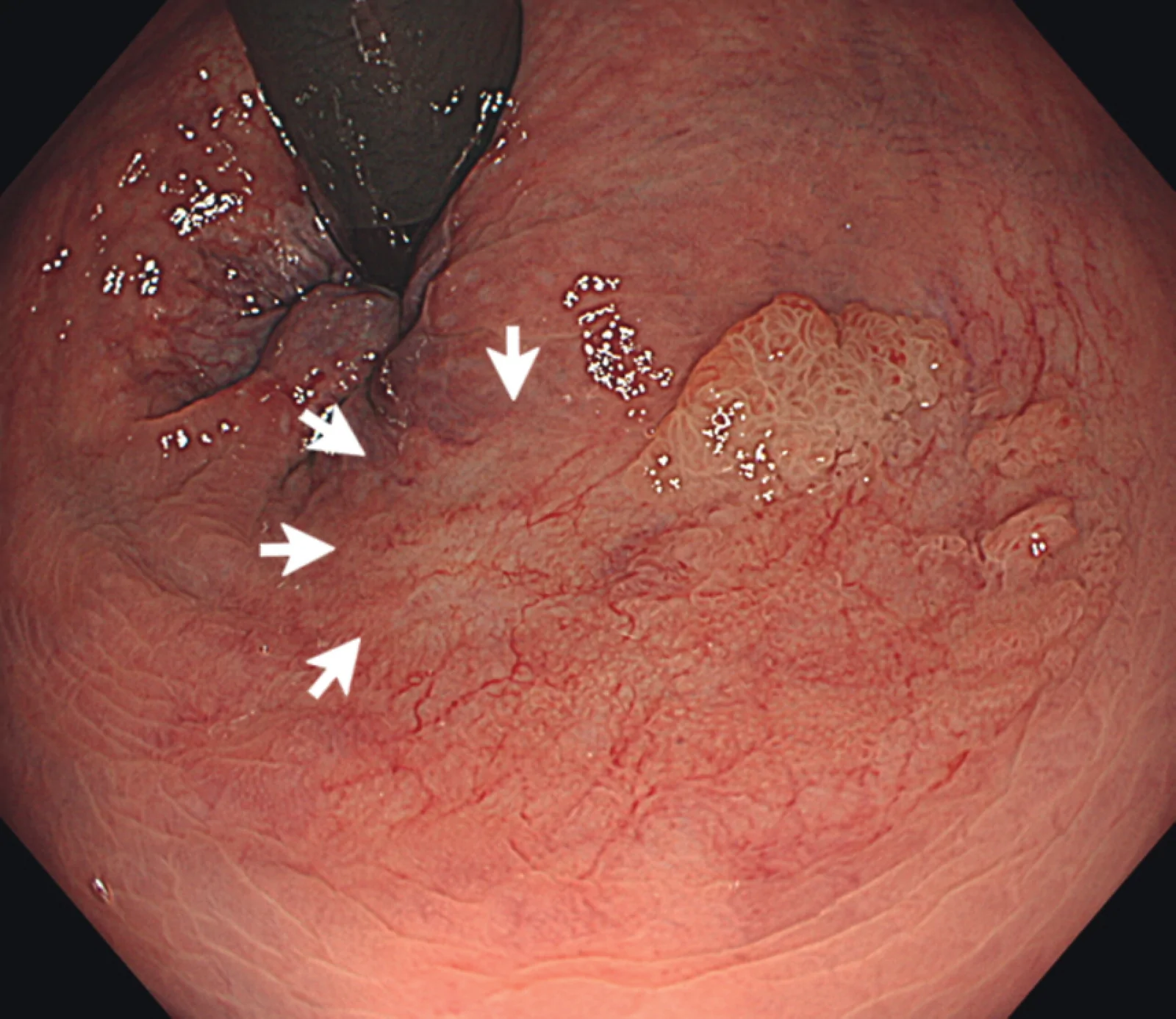
An endoscopic image of the lower rectum (Rb) showing a 24-mm type 0-IIa lesion. The white arrows indicate the scar from a previous hemorrhoidectomy.

**Fig. 2:**
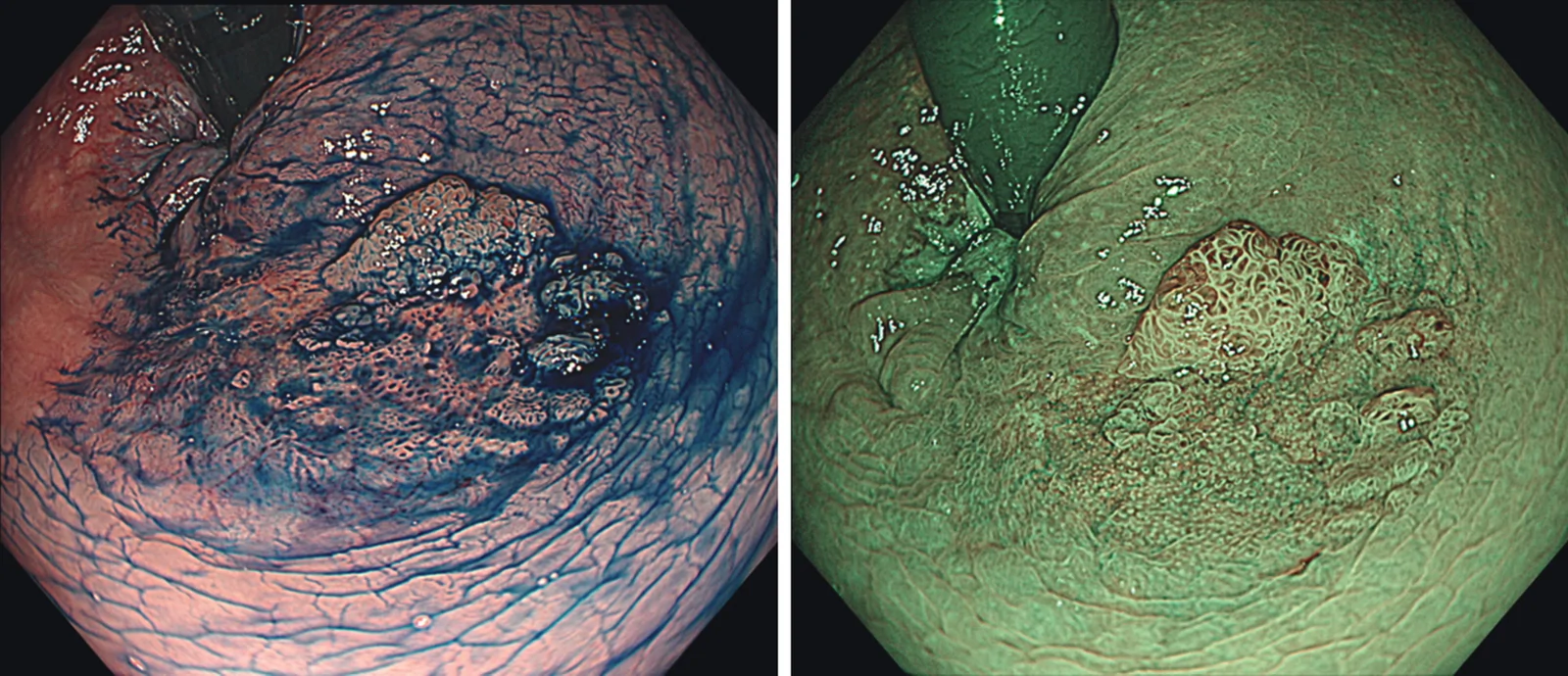
Indigo carmine chromoendoscopy ( **left** ) and conventional NBI (structure enhancement level A8) ( **right** ) demonstrate indistinct margins in the flat area of the lesion.

To overcome this, we applied the novel NBI+TXI mode ( Video 1 ). This technology integrates the structural enhancement and brightness correction of TXI with the vascular contrast of NBI, facilitating margin recognition. In this mode, the DL became clear, enabling accurate distinction of the true neoplastic margin from the surrounding skirt and fibrotic scar ( **Figs.** 3 and 4 ).

**Video 1** NBI+TXI mode clarified the indistinct margins of a rectal lesion on a post-hemorrhoidectomy scar, enabling successful en bloc resection with precise margins.


**Fig. 3:**
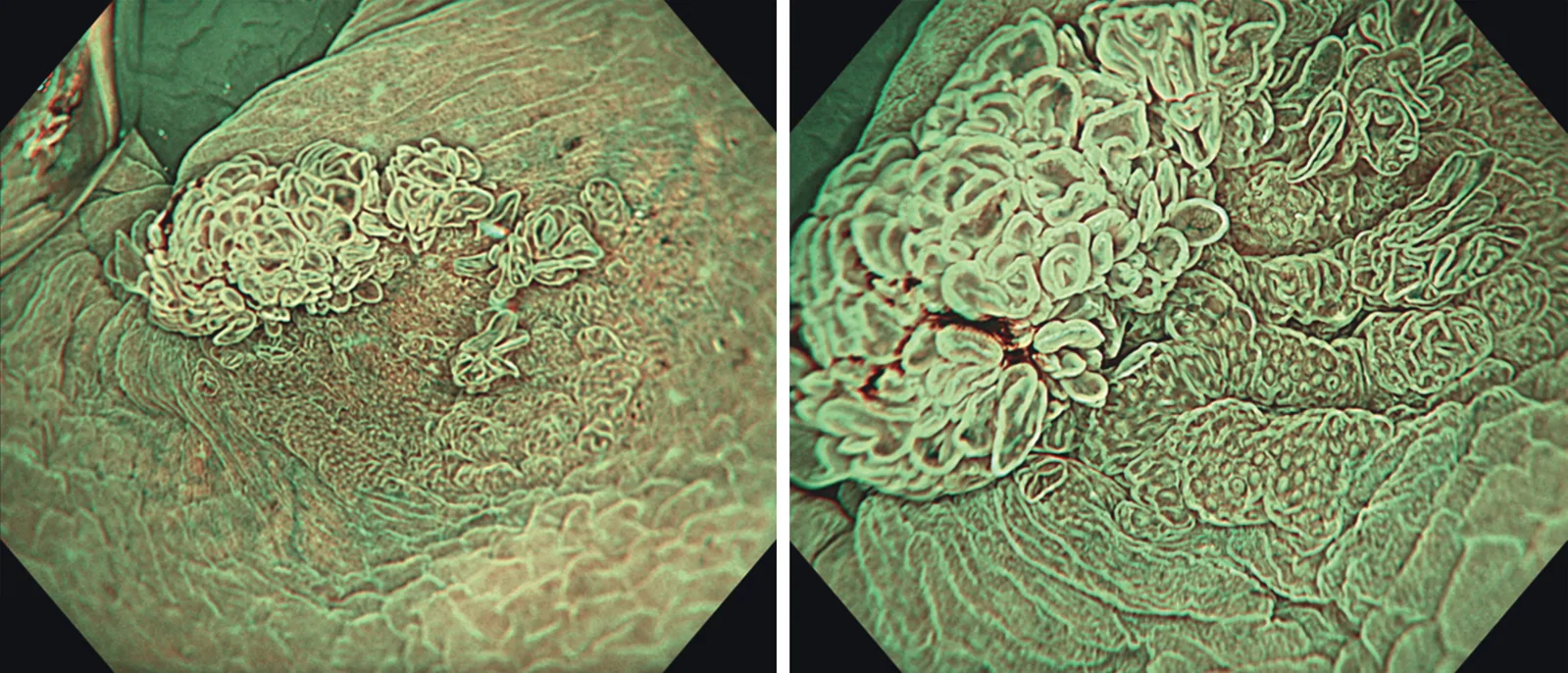
In the NBI+TXI mode, the margins of the flat area are more clearly visualized.

**Fig. 4:**
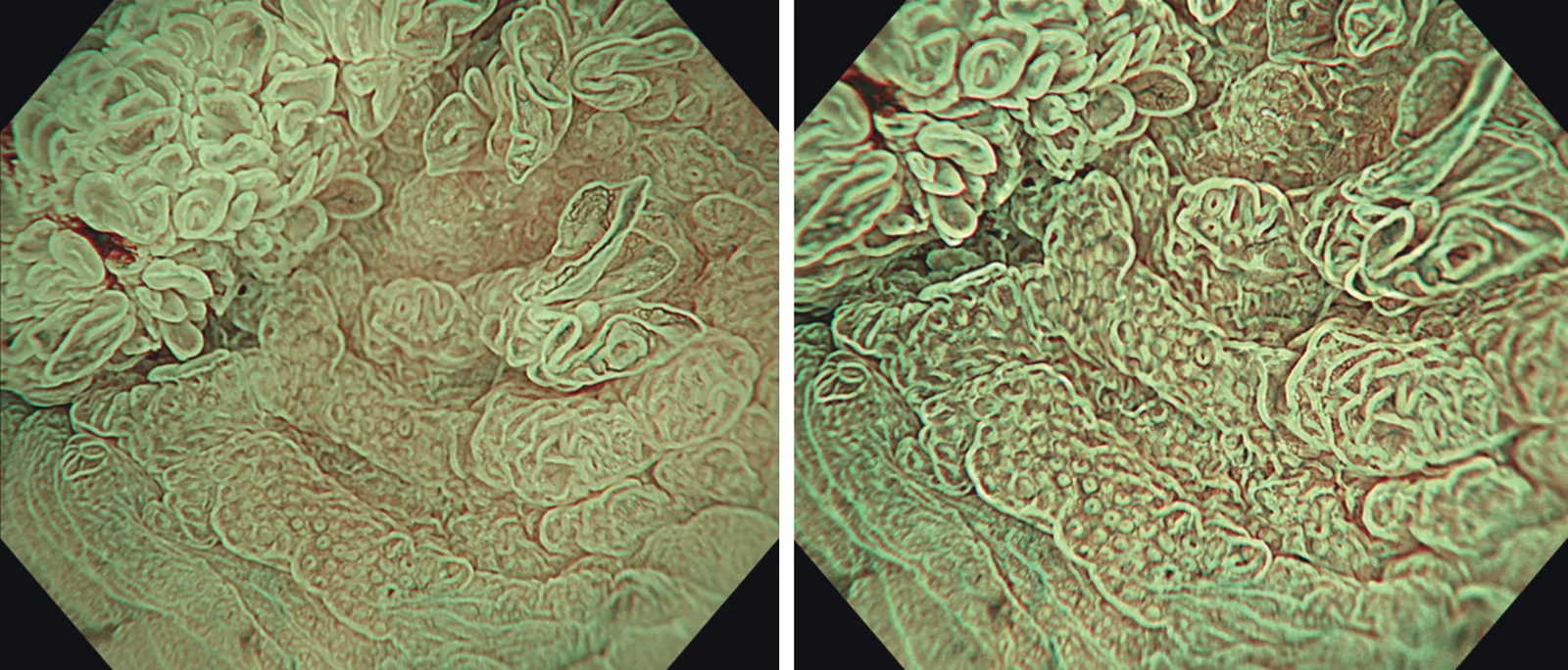
Magnified views of conventional NBI ( **left** ) and NBI+TXI mode ( **right** ; structure enhancement level A8). NBI+TXI facilitates easier recognition of the lesion margin.

Given the severe scar-induced submucosal fibrosis, we performed endoscopic submucosal dissection (ESD) with a partial incision of the inner circular muscle — an approach that allows for relatively safe resection of lesions over scars in the Rb 4​ — and achieved complete en bloc resection. Histopathological boundary determination remained difficult ( Fig. 5 ).

**Fig. 5:**
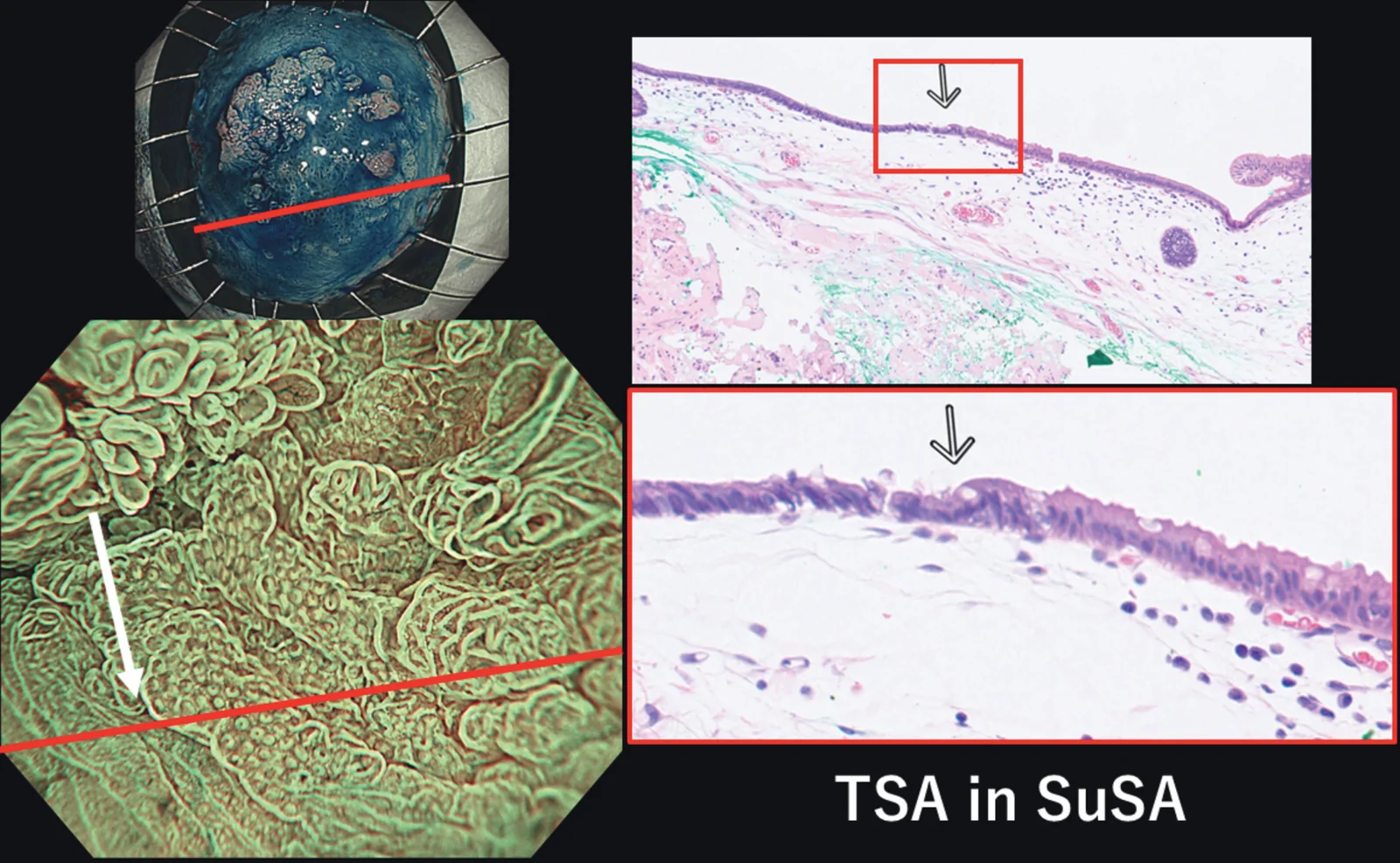
Histological mapping comparing the endoscopic and histopathological margins. Even on histopathological examination, the boundary between the normal mucosa and the lesion remains subtle.

In conclusion, although reports on the utility of NBI+TXI mode for margin diagnosis are lacking, this case suggests that it may be effective for evaluating lesions with unclear margins. Furthermore, ESD with partial inner circular muscle incision offers a safe therapeutic strategy for fibrotic scar lesions in the rectum. Further case accumulation is warranted.

Endoscopy_UCTN_Code_TTT_1AQ_2AB

## Declaration of GenAI Use

During the preparation of this work, the author(s) used Gemini in order to proofread and refine the English language of the manuscript. After using this tool/service, the author(s) reviewed and edited the content as needed and take(s) full responsibility for the content of the publication.
